# Impacts of Québec Primary Healthcare Reforms on Patients' Experience of Care, Unmet Needs, and Use of Services

**DOI:** 10.1155/2016/8938420

**Published:** 2016-02-10

**Authors:** Raynald Pineault, Roxane Borgès Da Silva, Sylvie Provost, Mylaine Breton, Pierre Tousignant, Michel Fournier, Alexandre Prud'homme, Jean-Frédéric Levesque

**Affiliations:** ^1^Direction de Santé Publique du Centre Intégré Universitaire de Santé et de Services Sociaux du Centre-Est-de-l'Île-de-Montréal, 1301 rue Sherbrooke Est, Montréal, QC, Canada H2L 1M3; ^2^Institut National de Santé Publique du Québec, 190 boulevard Crémazie Est, Montréal, QC, Canada H2P 1E2; ^3^Centre de Recherche du Centre hospitalier de l'Université de Montréal, 900 rue St-Denis, Montréal, QC, Canada H2X 0A9; ^4^Institut de Recherche en Santé Publique de l'Université de Montréal, 7101 avenue du Parc, Montréal, QC, Canada H3N 1X9; ^5^Faculté des Sciences Infirmières de l'Université de Montréal, 2375 chemin de la Côte-Ste-Catherine, Montréal, QC, Canada H3T 1A8; ^6^Centre de Recherche de l'Hôpital Charles-Lemoyne, 150 place Charles-Lemoyne, Bureau 200, Longueuil, QC, Canada J5C 2B6; ^7^Département des Sciences de la Santé Communautaire de l'Université de Sherbrooke, 3001 12^e^ avenue Nord, Sherbrooke, QC, Canada J1H 5H3; ^8^Department of Epidemiology, Biostatistics and Occupational Health, McGill University, 1020 avenue des Pins Ouest, Montréal, QC, Canada H3A 1A2; ^9^Centre for Primary Health Care and Equity, University of New South Wales, Kensington, NSW 2033, Australia; ^10^Bureau of Health Information, 67 Albert Avenue, Chatswood, NSW 2067, Australia

## Abstract

*Introduction*. Healthcare reforms launched in the early 2000s in Québec, Canada, involved the implementation of new forms of primary healthcare (PHC) organizations: Family Medicine Groups (FMGs) and Network Clinics (NCs). The objective of this paper is to assess how the organizational changes associated with these reforms have impact on patients' experience of care, use of services, and unmet needs.* Methods*. We conducted population and organization surveys in 2005 and 2010 in two regions of the province of Québec. The design was a before-and-after natural experiment. Changes over time between new models and other practices were assessed using difference-in-differences statistical procedures.* Results*. Accessibility decreased between 2003 and 2010, but less so in the treatment than in the comparison group. Continuity of care generally improved, but the increase was less for patients in the treatment group. Responsiveness also increased during the period and more so in the treatment group. There was no other significant difference between the two groups.* Conclusion*. PHC reform in Québec has brought about major organizational changes that have translated into slight improvements in accessibility of care and responsiveness. However, the reform does not seem to have had an impact on continuity, comprehensiveness, perceived care outcomes, use of services, and unmet needs.

## 1. Introduction

Most industrialized countries are facing efficiency problems in their healthcare systems associated with increased longevity and levels of morbidity among their populations. Issues with healthcare systems raise concerns since some countries, such as Canada and the United States, rank lower than other countries on most performance indicators, particularly those related to patients' experience of care, even though they devote comparable or even higher levels of financial resources [1].

To rectify the situation, different measures have been proposed such as implementing practices that are based on Patient-Centered Medical Home and Chronic Care Model concepts [2, 3]. These proposals recognize the widely accepted notion that primary care is the cornerstone in higher-performing healthcare systems [4, 5]. Search for solutions culminated in 2007 with the Institute for Health Improvement launching the Triple Aim Initiative [6]. The initiative promotes and supports projects aimed at achieving better population health and patients' experience of care and enhancing healthcare costs containment. The Canadian Foundation for Healthcare Improvement recently adopted the initiative, and some provinces, including Québec, are currently participating in a Triple Aim Collaborative [7].

Several components of the Triple Aim Initiative are found in the reforms initiated in Québec in the early 2000s. The aims of the reforms were to improve accessibility and continuity of primary healthcare and patients' experience of care while reducing unnecessary use of services, particularly among specialists services. The reforms also aimed to strengthen coordination of care in order to enable patients to navigate more easily the complexity of the healthcare system. Two new types of primary healthcare (PHC) organizations were created: Family Medicine Groups (FMGs) and Network Clinics (NCs) [8, 9]. FMGs incorporate elements of the Chronic Care and the Medical Home Models [3, 10, 11]. A typical FMG consists of 6 to 10 physicians working with nurses and providing services for 8,000 to 15,000 registered patients. FMGs, contracting with the Provincial Ministry of Health and Social Services, agree to increase services provision (e.g., extended opening hours and 24/7 phone access) in exchange for complementary public funding to support computerization and provide the means to hire additional staff, such as nurses. At onset of the reform, the target was to establish 300 FMGs in the province of Québec; as of 2010, there were 217 accredited FMGs. An FMG can comprise more than one clinical setting since it is based on physician participation and engagement, regardless of practice setting. Hence, an FMG can encompass more than one clinic with participating doctors. A complementary organizational model of PHC is the Network Clinic (NC), which was mainly implemented in Montréal under the initiative of the Regional Health Agency. Those clinics more specifically aim to foster accessibility through walk-in visits and provide access to diagnostic support, such as X-rays and lab tests, and to specialists. The distinction between FMG and NC is often blurred, as many clinics have both gained status and became FMG-NC. These clinics thus benefit from two sources of funding, provincial and regional.

In 2004, a local coordination structure was also created in Québec: 95 Health and Social Services Centres were entrusted by the Ministry of Health and Social Services to create local service networks [12]. The centres serve geographically defined populations. They were created by law, merging, on a geographical basis, long-term care hospitals, Local Community Services Centres, and, in most cases, acute care hospitals. Local Community Services Centres are public organizations created in the early 1970s under the governance of provincial authorities. They provide health and social services and physicians are generally paid on salary. Health and Social Services Centres were also mandated to increase collaboration among local actors and consequently to support the development of new emerging forms of local PHC organizations.

Structural changes and implementation processes associated with healthcare reforms have been well documented [13–16]. Likewise, outcomes associated with the introduction of innovative practices or new organizational models have been widely assessed [13, 14, 17–19]. However, the impact of PHC reforms on patients' experience of care and use of services at the population level has received much less attention [5, 18–21]. The Québec reform provided us with an opportunity to address that question. In close collaboration with the Regional Health and Social Services Agencies of Montréal and Montérégie, we conducted two studies in 2005 and 2010 to assess the evolution of PHC organizations and their impacts on patients' experience of care, unmet needs, and use of services in both regions [22, 23].

In a previous article, we discussed effects of the reforms on PHC organizations' changes [16]. Specifically, we found that significant organizational changes had resulted from the reform, mainly due to implementation of new types of PHC organizations (FMGs, NCs, and FMG-NCs). We also found that the main mediating factor accounting for these changes was the mimetic influence of exemplary PHC organizations that acted as promoters for implementation of the new models supported by provincial and regional policies.

The question raised in the conclusion of that article was whether these organizational changes had translated into improvement of patients' experience of care, unmet needs, and use of services. This is the issue we address in the current paper. More specifically, this paper aims to compare FMGs, NCs, and FMG-NCs implemented since 2003 with the other PHC organizations, in regard to the evolution of patients' experience of care, unmet needs, and use of services between 2003 and 2010.

## 2. Methods

### 2.1. Design

**Figure 1:**
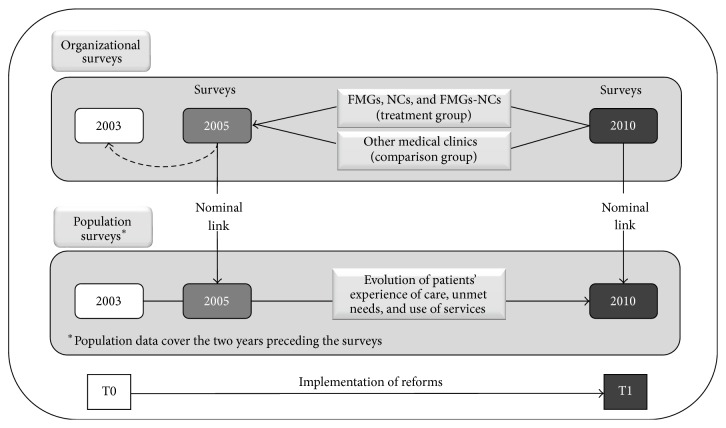
Study design.

The study design corresponded to a before-and-after natural experiment in which FMGs, NCs, and FMG-NCs constituted the treatment group, and the other clinics formed the comparison group (Figure 1). The intervention was the reforms initiated in 2003 as described in the Introduction. The study consisted of population and organization surveys carried out in 2005 and 2010 in the two most populous regions of the province of Québec: Montréal and Montérégie [23]. In both years, surveys were conducted on two independent population samples. They included 9,206 adults aged 18 or more in 2005 with a response rate of 64% and 9,180 adults in 2010 with a response rate of 56%. Concurrent surveys of all PHC organizations were also carried out; they included 659 organizations in 2005 with a response rate of 71% and 606 organizations in 2010 with a response rate of 62%. These surveys were preceded by telephone calls to the receptionists of all PHC practices to gather basic information such as type of practice (FMG, NC, and FMG-NC), number of physicians, presence of a nurse, and offering walk-in services.

The population samples were stratified but not proportionally with approximately 400 respondents in each of the 23 territories. Data were weighted to account for unequal selection probabilities resulting from this type of sampling. Poststratification weighting was applied for age and sex, based on 2006 and 2010 estimated Canadian census data. The population-based questionnaire focused on respondents' attachment to a PHC organization, healthcare and preventive services received, and experience of care in the two years preceding the surveys as well as unmet needs in the previous six months. The questionnaire drew mainly from two validated instruments, the Primary Care Assessment Survey and the Primary Care Assessment Tool, to which we added questions when a topic had not been addressed [24–26]. In addition to experience of care, the population questionnaire inquired about individual characteristics such as sex, age, education level, economic status, perceived health, and presence of morbidities [23].

The two organizational surveys included all PHC organizations in the two regions (659 in 2005 and 606 in 2010). In every organization, a key informant, usually the physician responsible for professional and administrative matters, completed the questionnaire. The organization questionnaire explored elements of vision, resources, structure, and practices [16]. Population and organization surveys were linked through respondents' identification of their regular source of primary care in the two years preceding the survey (2003–2005 and 2008–2010 corresponding to the T0 and T1 in Figure 1). Failing to identify a usual source of care, the respondent identified the PHC organization most frequently attended in the past two years. This organization was then regarded as the respondent's usual source of care.

### 2.2. Organizational Variables

PHC organizations were classified into two groups: a treatment group that included FMGs, NCs, and FMG-NCs; a comparison group that included Local Community Services Centres (LCSC) and Family Medicine Units (FMU) that were not FMG or NC, and all other clinics. Family Medicine Units are academic training units that are likely to reflect family medicine current practice and thus espouse hosting PHC organizations' dominant philosophy of care. In 2010, among the different PHC organization models in Montréal and Montérégie, group and solo practices were dominant, as they represented 73% of all organizations and were the usual source of care for 50% of all service users. They are privately run by physicians, who are paid on a fee-for-service basis. Costs are shared but not income. For the purposes of this study, FMGs implemented in Local Community Services Centres and Family Medicine Units were included in the treatment group. In the 2010 survey, there were only 11 LCSCs in the treatment group and 38 in the comparison group. The number of FMU remains marginal in the study as 9 were in the treatment group and only 3 in the comparison group.

Among the numerous indicators derived from responses to the organizational questionnaire, we retained the following (Appendix A):

- number of physicians;
- presence of nurses;
- number of information technologies used in the practice;
- blood taking services available in the building;
- radiology services available in the building;
- collaboration with other PHC clinics;
- collaboration with hospitals;
- predominant type of visits in the practice (walk-in, by-appointment, and mixed);
- scope of diagnostic, therapeutic, and preventive services offered.

At the time of the 2005 survey, 50 FMGs had already been implemented. Therefore, the baseline data were adjusted for those FMGs to take into consideration time elapsed between accreditation and the survey. We simply applied a regression analysis using pace of change of FMGs accredited after 2005 to those accredited before [16].

### 2.3. Experience of Care and Utilization-Related Variables

Patients' experience of care, unmet needs, and use of services were reported by population survey respondents. Experience of care variables were accessibility, continuity, comprehensiveness, responsiveness, and perceived care outcomes [27]. Operationalization details for these variables are presented in Appendix B.

We selected 28 indicators of experience of care and grouped them under five dimensions: accessibility (6 items), continuity (5 items), comprehensiveness (5 items), responsiveness (7 items), and perceived care outcomes (5 items). We carried out factor analyses within each of the five dimensions and calculated Cronbach's alpha on 2010 population data. Cronbach's alpha was over .60 for four of the five dimensions: continuity (.61), comprehensiveness (.79), responsiveness (.63), and care outcomes (.82). Cronbach's alpha was low for accessibility (.30), presumably reflecting the great heterogeneity of the indicators and the formative nature of this index [28]. In fact, the accessibility items are not highly correlated, which characterizes composite indices rather than reflective scales [29]. Experience of care variables were expressed as scores on a 10-point scale.

Aside from experience of care, which referred to the two years preceding the surveys, the population questionnaire contained information on use of emergency rooms, hospital admissions, visits to usual source of care, having a family doctor, reporting unmet needs, preventive care received, and sociodemographic characteristics of respondents [23]. Presence of unmet needs in the six months preceding the survey as well as utilization variables was dichotomous variables.

### 2.4. Data Analysis

To test for differences between 2003 and 2010 in the treatment and comparison groups, we applied the difference-in-differences technique and matched individuals with the propensity score method to ensure better comparability of the two groups [30–34]. This method is particularly well suited to compare change in outcomes over time between individuals exposed to an intervention (the treatment group) and individuals that are not (comparison group) [32]. Individuals were matched on the basis of propensity scores that estimate the probability that an individual with given characteristics is attached to a PHC organization of the treatment group [35]. The propensity score acts as a balancing score that renders the distribution of observed baseline covariates similar between the treatment group and the comparison group [36]. Analyses were carried out using STATA (version 13). Groups were compared after matching by propensity scores with *t*-tests. The results showed no significant *t* values, indicating that covariates were successfully balanced.

## 3. Results

### 3.1. Differences between Treatment and Comparison Groups on Selected Organizational Characteristics

As shown in Table 1, the treatment and comparison groups differ considerably with respect to the organizational characteristics presented, particularly in 2010.

Treatment group's PHC practices are larger and include virtually no solo practices. They are more likely to have nurses and use information technologies and to have blood taking and radiology services available in the building. They have also developed greater collaboration with other PHC practices and hospitals and offer a wider scope of diagnostic, therapeutic, and preventive procedures. Finally, a higher percentage of practices in the treatment group provide a mix of visits (walk-in and by-appointment) at the expense of other types of visits. This percentage increased between 2005 and 2010 from 27.3% to 47.1% whereas the percentage of those providing predominantly walk-in visits decreased from 24.0% to 9.9%. All other indicators followed the same trend, increasing between 2005 and 2010 in the treatment group but remaining more stable or even decreasing in the comparison group. For example, collaboration with other PHC practices decreased with 15.8% in the comparison group while it increased with 19.0% in the treatment group. We observe the same pattern for collaboration with hospitals, although the decrease was less in the comparison group (−7.1%).

### 3.2. Differences between Treatment and Comparison Groups in the Evolution of Patients' Experience of Care, Unmet Needs, and Use of Services

Table 2 shows the evolution of patients' experience of care, unmet needs, and use of services between 2003–2005 and 2008–2010, as well as differences in evolution between the two groups.

The most surprising results concern accessibility which declined between 2003–2005 and 2008–2010 in both groups. However, it declined to a lesser degree for individuals attached to practices of the treatment group than those of the comparison group. This result, in favor of the treatment group, is shown with the positive and significant DD (+0.18; *p* = 0.003) and corresponds to a 2.5% change. The percentage of change was calculated by dividing the DD value by the mean baseline scores of both groups. Indicators that contributed the most to this change were the possibility of seeing another doctor when the regular doctor was not available and adequate opening hours of the clinic (data not presented).

A positive DD value is also noted for responsiveness that, unlike accessibility, increased during the same period in the two groups but at a higher pace in the treatment group (+0.09; *p* = 0.031), corresponding to a 1.0% change. Indicators that contributed the most to this change are shorter waiting time between arrival at the clinic and seeing the doctor and courtesy of reception (data not presented).

Continuity generally improved between 2003–2005 and 2008–2010. At the two periods, the comparison group performed better, as shown by the negative and significant differences. The differences widened over the years but not enough to reach the level of statistical significance for the DD value (−0.16; *p* = 0.107). Indicators that contributed the most to the differences at the two periods are “how long the patient goes to his usual source of primary care” and “whether he always sees the same doctor at this place” (data not presented).

Perceived care outcomes scores slightly improved over time and more so in the comparison group. Although the difference between the two groups was significant in 2008–2010 (*p* < 0.01), the DD value failed to reach statistical significance (−0.10; *p* = 0.147).

The score for comprehensiveness was higher in the comparison than in the treatment group at the two time periods but especially in 2008–2010 (*p* = 0.002). Still, the change over time was not sufficient to yield a significant DD value. For all other indicators of experience of care, unmet needs, and use of services, DD values were not statistically significant.

## 4. Discussion

Implementation of FMGs in the two regions has brought about important organizational changes, mainly in terms of resources and structures of PHC organizations [16]. However, the impact of these organizational changes on patients' experience of care, unmet needs, and use of services has been so far limited. The major impact has been on accessibility. Introduction of the new PHC models has potentially slowed deterioration in accessibility. A second positive effect is on responsiveness. A main contributing factor to increased responsiveness was decreased waiting time between arrival of a patient in a doctor's office and the face-to-face encounter with the doctor. In that sense, this indicator of responsiveness partly mirrors accessibility.

The positive effect of FMGs and NCs on continuity has been less important than that of other PHC organizations. This result raises concerns because FMGs have been entrusted with improving continuity of care. Again, we should recall that main contributing factors to the relative decrease of continuity in the treatment group were not being able to always see the same doctor at the regular source of care and length of time a patient has been attached to the clinic. We further explored the lower increase in continuity in the treatment group by carrying out separate DD analyses for PHC organizations accredited between 2003 and 2006 and those accredited between 2007 and 2010. The DD values were very close in both analyses for all indicators except continuity which showed a negative and significant DD value for clinics of the treatment group accredited between 2007 and 2010 (data not presented). This difference could be explained partly by the fact that NCs which focus on accessibility more than continuity were mostly implemented during that period. An additional factor is that incentive-linked enrolment of patients presenting specific condition was implemented for all PHC organizations in 2003, thus lessening the advantageous position of FMGs and NCs [37].

Our findings for continuity contradict those reported by Tourigny et al. in their study of 1275 patients followed in five FMGs implemented in two regions of Québec [38]. That study found an increase in continuity but a decrease in accessibility after 18 months among the patients enrolled in those FMGs. Our study is not comparable to the Tourigny et al. study in many regards, namely, number of FMGs studied, inclusion of NCs as new forms of PHC organizations, and regions covered by the study [23, 38]. Our population sample size and the large number of PHC organizations surveyed allow us to generalize with a fair degree of confidence, at least for the two regions under study.

Like most studies that have examined continuity of care, our measures of continuity focus on face-to-face contacts with the regular doctor and not on group continuity (seeing different doctors or other professionals in the same clinic). This is presumably the reason why studies report solo practices ranking the highest in terms of patient experience of care, with the exception of accessibility [39]. This limitation in the measure of continuity has been raised by authors and new measures have been proposed to correct it [40, 41]. Finally, our results did not show that FMGs and NCs have had an impact on use of services or unmet needs.

Our results concur with results reported by other studies on the partial failure of FMGs and NCs in Québec to attain expected results. In a study involving the entire population of Quebec, Dunkley-Hickine found that a higher degree of physician participation in FMGs in a geographical area was not associated with improved accessibility for the population living in this area [42]. In a cohort study based on large administrative data banks and involving 79 FMGs implemented between 2002 and 2004, Strumpf observed no improvement in accessibility but a slight reduction in use of primary care and specialist services [43]. Using the same data bank, Héroux et al. followed a cohort of 122,722 patients enrolled in 79 FMGs compared to 675,102 who were not [44]. They found a small reduction in number of emergency visits for those in FMGs but, as in our study, no change in hospitalizations.

We must recognize that the PHC reform in Quebec has added resources: financial, technical, and human. However, the impact on the population has been limited. This contrasts, for example, with Ontario where the introduction of major financial incentives for physicians to adopt other modes of payment and particularly per capita and blended payment schemes has led to major changes in medical practice [9, 21, 45–47].

A survey conducted by the Commonwealth Fund in 2013 compared different countries and three Canadian provinces on several indicators of experience of care [48]. The report shows that experience of care reported by Quebec respondents, particularly regarding accessibility, was generally less favorable in Quebec than in Ontario, Alberta, and the rest of Canada. Since most of these indicators are related to PHC services, they reflect a relatively poor performance, given the major financial efforts devoted to reforms in this sector.

A recent report of the Auditor General of Quebec to the National Assembly drew a very critical picture of the situation around the implementation of FMGs and NCs [49]. It concluded that FMGs and NCs failed to fully attain the objectives set by the Ministry. It attributes this failure mainly to the absence of clear rules, guidelines, and incentives and to the lack of control of the Ministry and Regional Agencies over the implementation. This has left FMGs and NCs free to develop on their own without having to be fully accountable to the Ministry or the Regional Agencies.

A recent report of the C. D. Howe Institute by Forget has come to similar conclusions [50]. It underlines several factors that have slowed implementation of FMG policies, notably inadequate development of teamwork, and low registration rate of patients.

Overall, the results reported in this paper indicate that reforms in PHC organizations initiated in 2003 have brought about some expected benefits but have yet to be completed. The reported impact of the new types of PHC organizations on accessibility and responsiveness partly fulfills the objectives set at onset of the reforms. Other results raise concerns, notably regarding continuity, use of services, and unmet needs.

## 5. Limitations

Our study has limitations. A population survey lends itself to possible recall bias by respondents reporting on their experience of care. If present, the bias should be equally distributed among respondents. Recall bias is more likely to occur when referring to single events taking place at a given point in time than with an overall experience extending over a certain period of time.

We did not have information on nonrespondents to the population surveys, which could also introduce a bias. However, the bias was minimized by applying a weighting procedure that adjusts samples' data to the 2005 and 2010 populations' data. In addition, our analyses control for individual characteristics of respondents.

Measures of experience of care are expressed from the patient's points of view and perceptions. In that sense, these measures are subjective but still the most appropriate to describe experience of care. Our scales are largely derived from two validated instruments, PCAT and PCAS [24–26]. We constructed new scales of experience of care that were more adapted to our context. Following the classical theory of measure, we carried out factor analyses and calculated Cronbach's alpha coefficients. The coefficient values were generally close to the commonly accepted level (.70) except for accessibility which was low (.30) [51]. We have kept accessibility in our analyses, considering it is a formative composite index in which the items composing the index are not necessarily correlated nor substitutable with each other, as is the case in reflective scales [29].

The imputation method we applied to nonrespondents of the PHC organization survey might have introduced a bias since it is based on respondent data. As pointed out by Haziza, the nonresponse bias may be greater with nonresponse than with imputation, particularly when basic information is available, as was the case here from calls to receptionists prior to the organization survey [52]. We tested the magnitude of a possible bias by carrying out statistical analyses with respondent organizations only. The results obtained were similar to those with grouped respondents and nonrespondents [16].

## 6. Conclusion

In spite of important resources devoted to PHC reforms initiated in Quebec in the early 2000s, results so far have been limited in terms of experience of care improvement, reduction of utilization of services, and unmet needs. This is the conclusion we can draw when we compare the evolution of PHC practices that have become FMGs, NCs, or FMG-NCs to that of PHC practices that have not acquired such status. This is not to say that the healthcare system has not improved as a whole. For example, we observed that continuity has increased at the system level but more so in practices that were not FMG, NC, or FMG-NC. Accessibility has decreased but much less than it would have without FMGs, NCs, and FMG-NCs. These examples highlight the fact that it is hazardous and perhaps unfair to attribute specific effects of a social policy to a single intervention, be it success or failure. Social systems are complex and their components are highly interactive. The effect of a change may induce other, often unexpected, changes. Finally, implementing policy changes takes time [53]. This is the conclusion of a recent article that assessed one of the earliest and largest medical home pilots implemented in the United States [54]. How long it takes to realistically and fairly assess sustainable benefits brought about by primary care reforms such as the ones initiated in Québec in the early 2000s remains difficult to ascertain.

## Figures and Tables

**Table 1:** Percentage of PHC organizations with selected organizational characteristics, in comparison and treatment groups, 2005 and 2010 surveys.

	Comparison group			Treatment group		
	(other medical clinics)			(FMG-NC, FMG, NC)		
	*n* = 420			*n* = 121		
	2005	2010	Diff.	2005	2010	Diff.
	%	%		%	%	
Size of practice (number of physicians)						
1	39.3	41.4	2.1	4.1	4.1	0.0
2 to 3	23.3	24.0	0.7	15.7	14.0	−1.7
4 to 6	19.0	16.9	−2.1	19.0	14.9	−4.1
7 or more	18.4	17.7	−0.7	61.2	67.0	5.8
Presence of nurses						
Yes	26.2	30.7	4.5	68.6	87.6	19.0
Information technologies used in the practice						
1 or more	50.0	60.0	10.0	80.2	92.5	12.3
Blood taking service available in the same building						
Yes	40.7	40.7	0.0	66.9	73.6	6.7
Radiology available in the same building						
Yes	6.2	7.4	1.2	22.3	28.9	6.6
Collaboration with other PHC practices						
Yes	41.0	25.2	−15.8	58.7	77.7	19.0
Collaboration with hospitals						
Yes	43.8	36.7	−7.1	60.3	77.7	17.4
Type of visits in the practice predominantly						
Walk-in	19.5	13.8	−5.7	24.0	9.9	−14.1
By-appointment	66.5	70.5	4.0	48.7	43.0	−5.7
Mixed	14.0	15.7	1.7	27.3	47.1	19.8
Quantity of diagnostic and therapeutic services available in the practice						
Several (8 out of 10 or more)	12.6	8.3	−4.3	36.4	39.7	3.3

**Table 2:** Difference-in-differences (DD) of experience of care, unmet needs, and utilization of services (%) between users having NC, FMG, or FMG-NC as usual source of care and those having another type of medical clinic, 2003–2005 and 2008–2010.

	2003–2005				2008–2010				DD	% of change	*p*
	Comparison group	Treatment group	Diff.	*p*	Comparison group	Treatment group	Diff.	*p*			
	(other medical clinics)	(FMG-NC, FMG, NC)			(other medical clinics)	(FMG-NC, FMG, NC)					
	(*n* = 3135)	(*n* = 3063)			(*n* = 3135)	(*n* = 3063)					
Accessibility of services (max score: 10)	7.20	7.25	0.05	.252	6.75	6.98	0.23	<.001	0.18	2.5	.003
Continuity (max score: 10)	8.16	8.00	−0.16	.002	8.65	8.33	−0.32	<.001	−0.16	−2.0	.107
Comprehensiveness (max score: 10)	8.51	8.43	−0.08	.106	8.42	8.27	−0.15	.002	−0.07	−0.8	.343
Responsiveness (max score: 10)	8.71	8.62	−0.09	.004	8.86	8.86	0.00	.031	0.09	1.0	.031
Perceived care outcomes (max score: 10)	8.58	8.50	−0.08	.105	8.75	8.57	−0.18	<.001	−0.10	−1.2	.147
% of users who reported unmet needs for care	19.1	18.8	−0.30	.777	16.1	16.4	0.30	.691	0.6	3.2	.632
% of users who attended usual source of care six times or more	22.8	24.2	1.4	<.001	19.9	19.3	−0.6	.607	−2.0	−8.5	.200
% of users who attended emergency room at least once	34.7	36.7	2.0	.101	35.2	36.2	1.0	.396	−1.0	−2.8	.545
% of users hospitalized at least once	16.1	16.5	0.4	.681	17.8	19.8	2.0	.105	1.6	9.8	.413

**Table 3:** (Appendix A) Construction of organizational indicators.

Size of practice	
*How many general practitioners, including those working part time, currently work at your clinic?*	
Presence of nurses	
*How many nurses currently work at your clinic?*	
Information technologies used in the practice	
In your clinic, do you use…	
*access to the health and social services telecommunications network?*	
*a Web-based appointment system for patients?*	
*computerized tools for continuing professional education?*	
*electronic medical records?*	
*electronic interface to diagnostic imaging laboratory services?*	
*computerized tools to aid medical decision-making?*	
Blood taking service available in the same building	
Are the following services available in the building where your clinic is located…	
*Blood samples taking?*	
Radiology available in the same building	
Are the following services available in the building where your clinic is located…	
*Radiology?*	
Collaboration with other PHC practices	
Does your clinic have formal or informal arrangements with other PHC clinics…	
*to schedule services offered? *	
*to access to technical services? *	
*to exchange resources? *	
Collaboration with hospitals	
Does your clinic have formal or informal arrangements with hospitals…	
*to schedule services offered?*	
*to access to technical services? *	
*to exchange resources? *	
Predominant type of visits in the practice	
What percentage of walk-in visits to all visits do you provide at your clinic?	
*0% to 25% = *By-appointment visits	
*26 to 75% = *Mixed	
51% or more = Walk-in visits	
Quantity of diagnostic or therapeutic services available in the practice	
At your clinic, are the following services available…	
*Strep-test?*	
*Skin biopsy?*	
*IUD insertion?*	
*Musculo-skeletal injection/aspiration?*	
*Suture/minor surgery?*	
*Cervical smear (Pap test)?*	
*Childhood vaccination?*	
*Influenza vaccination?*	
3 or less among these = Few	
4 or 5 among these = A fair number	
6 or more among these = Several	

**Table 4:** (Appendix B) Construction of experience of care indices.

The following questions refer to the usual PHC source identified by the respondents	
Accessibility of services	
if the doctor who is responsible for your care is not available, you can see another doctor?	0. Never/Sometimes/Often
	1. Always
how long does it take to see the doctor by appointment?	0. Two weeks or more
	1. Less than two weeks
how long does it usually take to get there?	0.15 minutes or more
	1. Less than 15 minutes
the office hours are convenient?	0. Not at all/A little/Somewhat agree
	1. Strongly agree
it is easy to reach someone by telephone to make an appointment?	0. Not at all/A little/Somewhat agree
	1. Strongly agree
it is easy to talk to a doctor or nurse by telephone?	0. Not at all/A little/Somewhat agree
	1. Strongly agree
Continuity of care	
you see the same doctor?	0. Never/Sometimes/Often
	1. Always
how long have you been going there?	0. Five years or less
	1. More than 5 years
your medical history is known?	0. Not at all/A little/Somewhat agree
	1. Strongly agree
they are aware of all the prescribed drugs you take?	0. Not at all/A little/Somewhat agree
	1. Strongly agree
you can receive routine ongoing care for a chronic problem?	0. Not at all/A little/Somewhat agree
	1. Strongly agree
Responsiveness	
how long do you have to wait between the scheduled time of appointment and the time you actually see the doctor?	0. Less than 60 minutes
	1. 60 minutes or more
the staff answer your questions clearly ?	0. Not at all/A little/Somewhat agree
	1. Strongly agree
you feel respected?	0. Not at all/A little/Somewhat agree
	1. Strongly agree
you are greeted courteously at the reception?	0. Not at all/A little/Somewhat agree
	1. Strongly agree
your physical privacy is respected?	0. Not at all/A little/Somewhat agree
	1. Strongly agree
the doctors spend enough time with you?	0. Not at all/A little/Somewhat agree
	1. Strongly agree
the local of the clinic are pleasant?	0. Not at all/A little/Somewhat agree
	1. Strongly agree
Comprehensiveness	
all your health problems are taken care of whether they are physical or psychological?	0. Not at all/A little/Somewhat agree
	1. Strongly agree
the doctor takes the time to talk to you about prevention and asks you about your lifestyle habits?	0. Not at all/A little/Somewhat agree
	1. Strongly agree
they help you get all the health care services you need?	0. Not at all/A little/Somewhat agree
	1. Strongly agree
your opinion and what you want are taken into account in the care that you receive?	0. Not at all/A little/Somewhat agree
	1. Strongly agree
you are given help to weigh the pros and cons when you have to make decisions about your health?	0. Not at all/A little/Somewhat agree
	1. Strongly agree
Outcome of care	
the services you get help you to better understand your health problems?	0. Not at all/A little/Somewhat agree
	1. Strongly agree
the services you get help you to prevent certain health problems before they appear?	0. Not at all/A little/Somewhat agree
	1. Strongly agree
the services you get help you to control your health problems?	0. Not at all/A little/Somewhat agree
	1. Strongly agree
the professionals you see encourage you to follow the treatments prescribed?	0. Not at all/A little/Somewhat agree
	1. Strongly agree
the professionals you see help motivate you to adopt good lifestyle habits?	0. Not at all/A little/Somewhat agree
	1. Strongly agree
Unmet needs	
During the last 6 months, did you feel you needed to see a doctor for a health problem but didn't see one?	0. No
	1. Yes

## Appendices

### A. Organizational Variables

See Table 3.

### B. Experience of Care Indices

See Table 4.
